# Governance configurations of coordination in municipal health promotion: insights from an egocentric network analysis

**DOI:** 10.1186/s12913-026-15347-8

**Published:** 2026-08-22

**Authors:** Hanna Richter, Tatiana Mamontova, Lisa Kühne

**Affiliations:** SOCIUM – Research Center on Inequality and Social Policy, Department Health, Long-term care and Pensions, University Bremen, Mary-Somerville-Straße 5, 28359 Bremen, Germany

**Keywords:** Network governance, Coordination capacity, Boundary spanning, Social network analysis, Municipal health promotion, Intersectoral collaboration

## Abstract

**Background:**

Municipal health promotion requires coordination across organizational and sectoral boundaries. Fragmented responsibilities, strong organizational autonomy and limited formal authority can make sustained cross-sectoral coordination challenging. Although research on Health in All Policies and governance capacity has highlighted the organizational conditions necessary for coordination, little is known about how coordination functions are embedded within municipal governance networks. This study examines the structural conditions that shape cross-sectoral coordination in municipal health promotion.

**Methods:**

Egocentric social network analysis was used to map the professional networks of 14 health professionals working in primary schools and neighborhood settings in a single German municipality. Data were collected through semi-structured interviews combined with structured network elicitation, capturing 662 individuals, their reported interconnections and organizational attributes. Network measures of structural integration, modular differentiation, local cohesion and relational dependency were combined with modularity analysis and ego-removal comparisons. Qualitative interview data contextualized the structural findings and supported the functional interpretation of network modules.

**Results:**

The networks varied considerably in terms of the structural organization of cross-sectoral coordination. Modularity analysis revealed the following recurring governance domains: education; neighborhoods and social spaces; professional coordination and peer networks. The combined analysis identified three governance configurations. In ego-dependent integration, internally cohesive network areas remained intact after the removal of the ego, but lost connections to other areas. In substitutable coordination, connectivity persisted through alternative individual or collective coordination hubs or relational pathways. In distributed integration, cross-module connectivity was supported by multiple relationships and remained largely intact even without the focal actor. Therefore, network connectedness and local cohesion alone did not determine how strongly coordination depended on individual brokers.

**Conclusion:**

Municipal coordination capacity depends not only on coordinating professionals and their resources, but also on how brokerage and cross-sectoral relationships are structurally embedded in governance networks. Therefore, strengthening municipal health promotion requires the establishment of adequately resourced and formally supported coordination roles, as well as investment in collective coordination arenas, alternative coordination pathways, and direct cross-sectoral relationships. Recognizing network work as a continuous strategic function can help to create governance structures that are less vulnerable to the loss of individual actors.

**Supplementary Information:**

The online version contains supplementary material available at 10.1186/s12913-026-15347-8.

## Introduction

The Ottawa Charter established an understanding of health that emphasizes its social dimension. Health is created in people’s everyday lives, whether it be where they live, learn, work or spend their leisure time [1]. Based on this broader understanding, the Health in All Policies (HiAP) approach aims to systematically incorporate health considerations into decision-making processes in all policy areas. This is particularly relevant at the municipal level, where sectors such as healthcare, education, social services, urban planning, and the environment intersect. However, municipal governance is highly fragmented. Responsibilities and resources are distributed across organizations with different legal mandates, professional logics, and political priorities [2–4]. As a result, both overlap and underlap frequently occur: multiple organizations may share responsibility for the same issue, while other responsibilities remain unclear or are not assumed by any organization [5]. This can result in gaps in preventive services and coordination that depends on informal negotiation rather than clear hierarchical authority [1, 6]. The implementation of HiAP therefore depends on governance arrangements capable of integrating responsibilities distributed across organizational and sectoral boundaries.

Governance refers to the coordination of collective action among interdependent actors from the public, private, and civil sectors whose resources, competencies, and responsibilities are distributed across organizational boundaries and cannot be coordinated through hierarchical steering alone [7, 8]. From this perspective, inter-organizational networks have become a key form of public governance. Network governance enables autonomous organizations to coordinate collective action through relationships, information exchange, and shared decision-making, rather than through formal authority. Depending on factors such as trust, network size, goal consensus and task complexity, governance networks may adopt different organizational forms, including shared-governance, lead organizations governance and network administrative governance. These governance arrangements provide different structural conditions for cross-sector coordination [9].

However, the existence of a network-governance alone does not guarantee effective coordination. The extent to which governance arrangements enable organizations to coordinate activities, integrate resources, and address shared challenges varies. This structural potential is commonly referred to as governance capacity. Although there is no universally accepted definition, the term ‘governance capacity’ is generally understood to refer to the potential of a governance system. Building on Morgan’s [10] conceptualization of capacity as characteristics enabling the creation of developmental value and Fukuyama’s [11] view of capacity as a measure of inputs rather than performance, van Popering-Verkerk et al. [12] define governance capacity as a system’s potential to coordinate actors and resources in tackling collective challenges. In this study, governance capacity is understood specifically as the structural conditions that enable or constrain cross-sectoral coordination within local governance networks.

Although cross-sector collaboration is widely acknowledged as essential for implementing HiAP, existing research has primarily focused on collaborative processes and organizational factors that influence coordination, such as mandates, resources and professional logics. In contrast, the structural configurations of municipal governance networks that shape conditions for cross-sector coordination have received considerably less attention. To address this gap, this study uses Social Network Analysis to compare governance configurations across municipal health networks, examining the following research question: Which governance configurations provide favorable structural conditions for cross-sector coordination?

## Methods

This study takes a structural approach to examining municipal governance networks and the conditions under which cross-sector coordination can emerge. Rather than evaluating the outcomes of collaboration, the analysis focuses on the relational structures through which coordination is enabled, constrained or dependent on specific actors.

### Study design

This study employs a comparative ego-network design to analyses variations in the governance structures. The data are based on 14 egocentric network maps of health professionals working in primary schools and local communities within a single German municipality. The sample was selected using purposive sampling guided by principles of data saturation [13], enabling in-depth analysis while ensuring sufficient variation and volume management. Focusing on multiple neighborhood networks within a single municipal context enables the analysis of structural differences under comparable institutional conditions. Germany is a particularly relevant setting for this study, as cross-sector health promotion is embedded within a highly fragmented governance system. In this system, responsibilities are distributed across autonomous organizations, and municipalities are given discretion over local collaboration [3]. Inter-organizational networks play a central role in this context, making it an ideal case study for examining how governance configurations shape the structural conditions for coordination.

The ego-network approach was chosen because municipal health professionals occupy coordinating positions within local governance structures and have in-depth knowledge of the organizations involved in neighborhood-based health promotion. Each ego-network therefore represents the local governance structure surrounding a health professional, including the relationships between organizations within it.

### Data collection

The interviews combined a semi-structured qualitative interview with a structured network elicitation procedure, with both components administered within a single session. Although the network data form the main basis for the analysis in this study, selected qualitative interview excerpts are used to provide context for interpreting the structural governance findings. Participants were purposively selected because their professional roles involve building and coordinating interorganizational networks within municipal health promotion. During recruitment, efforts were made to ensure diversity in terms of professional background, years of experience, and field of activity. The interviews, which were audio-recorded and transcribed verbatim in accordance with the guidelines of Dresing and Pehl [14], lasted between 31 and 47 min and were conducted between July 2023 and March 2024.

Network data were collected using the software Vennmaker, a free tool for visual network generation. As an ego-centered design, each network reflects the actor’s perspective on relevant relations. Following the Kahn and Antonucci [15] concentric circle model, respondents were asked to identify individuals, institutions, or task groups with whom they collaborate in their professional context, resulting in a total of 662 identified alters across all interviews. For each alter, additional attributes (e.g., organizational affiliation, role, or sector) and inter-alter relations were recorded. This approach allows ego perceptions and structural tendencies to be identified across networks, enabling intra- and interpersonal comparisons to be made during the evaluation [16]. Ethical approval was obtained from the University of Bremen, and all participants provided informed, written consent.

### Data analysis

The network data were processed and analyzed using Gephi 1.0. The analysis proceeded in three steps. First, network measures were calculated and compared across all 14 ego networks [17]. The measures were organized into four analytical dimensions. *Structural integration* was examined using network size, average degree, edge density, and average path length. *Modular differentiation* was assessed using modularity, indicating the extent to which networks were divided into internally connected subgroups. *Local cohesion* was examined using global and local clustering coefficients and the number of triangles. Finally, *relational dependency* was assessed using ego betweenness centrality, indicating the extent to which the focal health professional occupied an intermediary position between otherwise weakly connected parts of the network. The selection of measures was guided by Marsden [18], with emphasis on structural characteristics for which egocentric network data provide comparatively robust approximations of the underlying network structure, particularly degree-based measures and betweenness centrality.

Secondly, a modularity analysis was performed to identify structurally coherent modules within each ego-network. These modules were examined in terms of their organizational composition and interpreted according to their dominant functional roles, in order to identify recurring governance domains across networks. Additionally, ego removal was employed to evaluate the extent to which these modules remained structurally connected or became disconnected, thereby offering insights into the dependence of cross-module integration on the focal health professionals. The modularity resolution was set to 1.0, which is appropriate for detecting fine-grained subgroup structures in small ego-centered network [19].

Third, three contrasting networks were selected for closer comparative analysis based on differences in the configuration of *structural integration*,* modular differentiation*,* local cohesion*, and *relational dependency*. The comparative analysis combined the four analytical dimensions and the ego-removal comparison. This enabled us to examine how different structural characteristics co-occurred. We also assessed whether cross-module integration was distributed evenly across alternative relationships or concentrated in the intermediary position of the focal actor.

The network visualizations were created using the ForceAtlas2 layout algorithm. This algorithm arranges nodes by simulating the physical forces of their relational proximity. The visualizations illustrate the modularity analysis and the structural patterns of network measures, both with and without the ego node. They demonstrate whether modules remain connected or become separated when the focal actor is removed, thereby supporting the interpretation of structural dependence on the ego.

## Results

### Structural variation across governance networks

The structural indicators revealed considerable variation across the 14 ego networks (see Table 1). In regard to *structural integration*, the networks varied considerably in terms of size, density and average degree. Network size ranged from 29 to 64 actors, edge density from 0.093 to 0.303, and average degree from 4.83 to 11.80. Average path lengths showed less variation, ranging from 2.07 to 2.92, indicating that actors were generally connected through a small number of intermediaries. *Modular Differentiation* also varied across the sample. Modularity values ranged from 0.373 to 0.579, indicating differences in the extent to which the networks were divided into internally connected subgroups. *Local cohesion* showed greater variation, with global clustering coefficients ranging from 0.283 to 0.717. There was also considerable variation in the number of triangles between networks, indicating substantial variation in the prevalence of closed triadic structures. Finally, *relational dependency* differed considerably across the networks. The ego betweenness centrality score ranged from 0.241 to 0.762, indicating the extent to which the health professionals in question occupied intermediary positions within their respective networks.

**Table 1:** Network characteristics of ego-centered networks

Participant	Network Size	Average Degree	Edge density	Average path length	Global clustering coefficient	Modularity	Betweenness centrality	Local clustering coefficent	Number of triangles
Ego 1	53	4,83	0,093	2,495	0,424	0,373	0,437	0,033	11
Ego 2	49	6,776	0,141	2,715	0,451	0,445	0,379	0,132	18
Ego 3	41	8,293	0,207	2,761	0,375	0,417	0,278	0,054	5
Ego 4	38	6,0	0,162	2,462	0,656	0,452	0,539	0,105	18
Ego 5	40	11,8	0,303	2,073	0,716	0,419	0,495	0,174	66
Ego 6	42	7,33	0,179	2,281	0,517	0,423	0,523	0,08	11
Ego 7	38	6,526	0,176	2,424	0,596	0,485	0,317	0,12	11
Ego 8	44	5,591	0,13	2,496	0,717	0,579	0,762	0,03	10
Ego 9	61	7,639	0,127	2,468	0,647	0,476	0,497	0,062	29
Ego 10	45	5,067	0,115	2,923	0,397	0,455	0,458	0,065	10
Ego 11	29	6,897	0,246	2,723	0,556	0,482	0,365	0,218	12
Ego 12	55	10,291	0,191	2,315	0,539	0,405	0,55	0,107	64
Ego 13	64	6,321	0,1	2,678	0,438	0,433	0,241	0,08	18
Ego 14	63	8,762	0,141	2,788	0,283	0,444	0,403	0,116	35

The descriptive correlations revealed two main patterns. Firstly, networks with stronger connections tended to exhibit greater local relational redundancy. The average degree was positively associated with edge density (*r* = 0.73) and the number of triangles (*r* = 0.85), while edge density was associated with local clustering (*r* = 0.72). This suggests that networks with greater connectivity also contain more overlapping and locally interconnected relationships. Secondly, ego betweenness centrality was largely unrelated to edge density and average degree, indicating that greater overall connectedness does not necessarily correspond to lower dependency on the health professional as an intermediary. However, ego betweenness was moderately associated with global clustering (*r* = 0.59) and modularity (*r* = 0.44). This suggests that, even in cohesive and modularly differentiated networks, the ego may be necessary to connect different network areas. Given the small number of networks, these correlations are interpreted descriptively rather than inferentially.

Overall, the findings demonstrate that governance networks vary across all four dimensions, but not uniformly. Notably, structural integration and local cohesion do not necessarily equate to low relational dependency. This variation is key to the subsequent analysis of governance domains and configurations.

### Functional domains of cross-sector governance

The modularity analysis identified distinct subgroups within each ego network. Based on their organizational composition and functional orientation, these modules were categorized into recurring governance domains. Despite structural variation between the 14 networks, three domains were present in most cases: education; professional coordination and peer exchange. The strength and pattern of connections within and between these domains varied across networks.

The *education domain* included educational institutions and professionals involved in education, such as early childhood education centers, schools, school social workers, psychologists, and regional advisory and support services. It also included non-school educational providers, such as community education programmers and adult learning institutions. This domain brought together actors working at the intersection of educational and health-related responsibilities. The *professional coordination and peer exchange domain* included health professionals working in schools and neighborhoods, as well as coordinating organizations, such as public health services. These modules connected individuals involved in professional exchange, joint planning and coordination across the health, education and social sectors. The *neighborhood and social space domain* comprised organizations such as family and neighborhood centers, youth services, welfare organizations, and local social organizations. These modules reflected the organization of prevention and health promotion activities within everyday living environments.

In addition to these recurring domains, individual networks contained more specialized modules related to specific local priorities or areas of professional activity, such as Health Care Service or Health Promotion. Tables A1 and A2 in the Appendix provide detailed information on module composition and distribution across ego-networks.

### Governance configurations

The network-level analysis revealed that the combination of structural integration, *modular differentiation*, *local cohesion* and *relational dependency* varies across the observed governance networks. To examine how these structural characteristics, interact within individual networks, three contrasting configurations were selected for closer analysis. This comparison uses a combination of network measures and ego-removal analysis to determine whether the connections between modules are structurally embedded within the network or if they primarily depend on the intermediary position of the focal health professional. Table 2 provides a detailed explanation of the color coding of modules in the network visualizations.

**Table 2:** Colour legend for network figures

Modul Colour	Modul
Pink	Educational Module
Blue	Professional Coordination and Peer Network Module
Green	Neighbourhood and Social Space Module
Orange	Health Care Service Module
Red	Health Promotion and Prevention Module
Black	Cross Sector Module

**Fig. 1:**
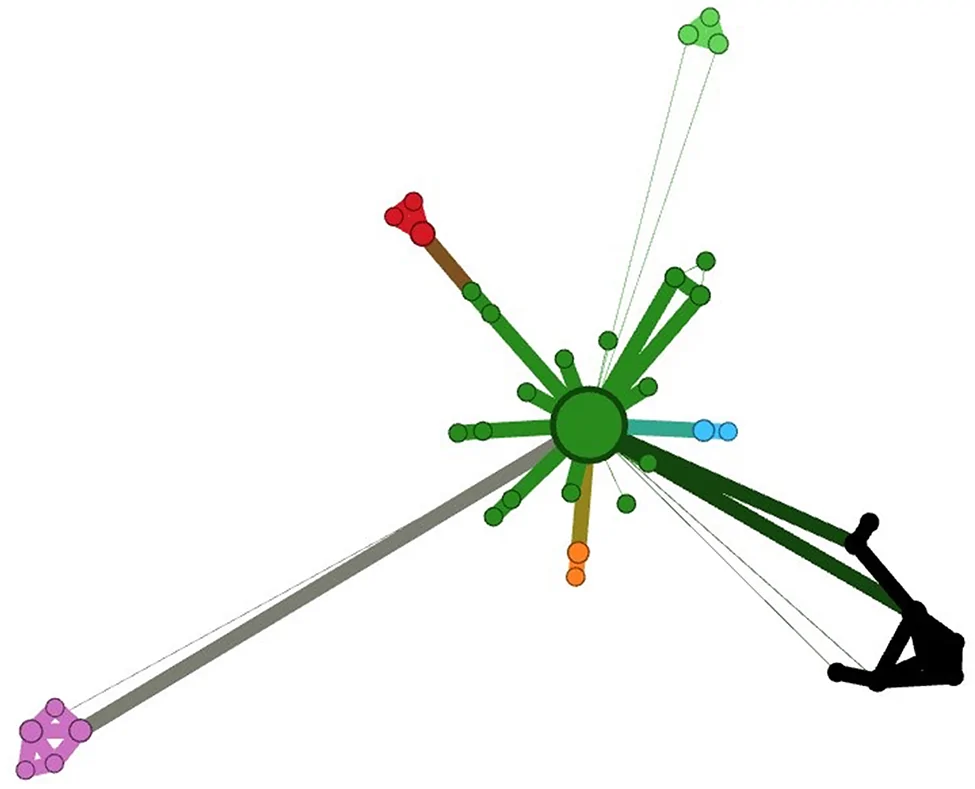
Ego-dependent governance configuration

**Fig. 2:**
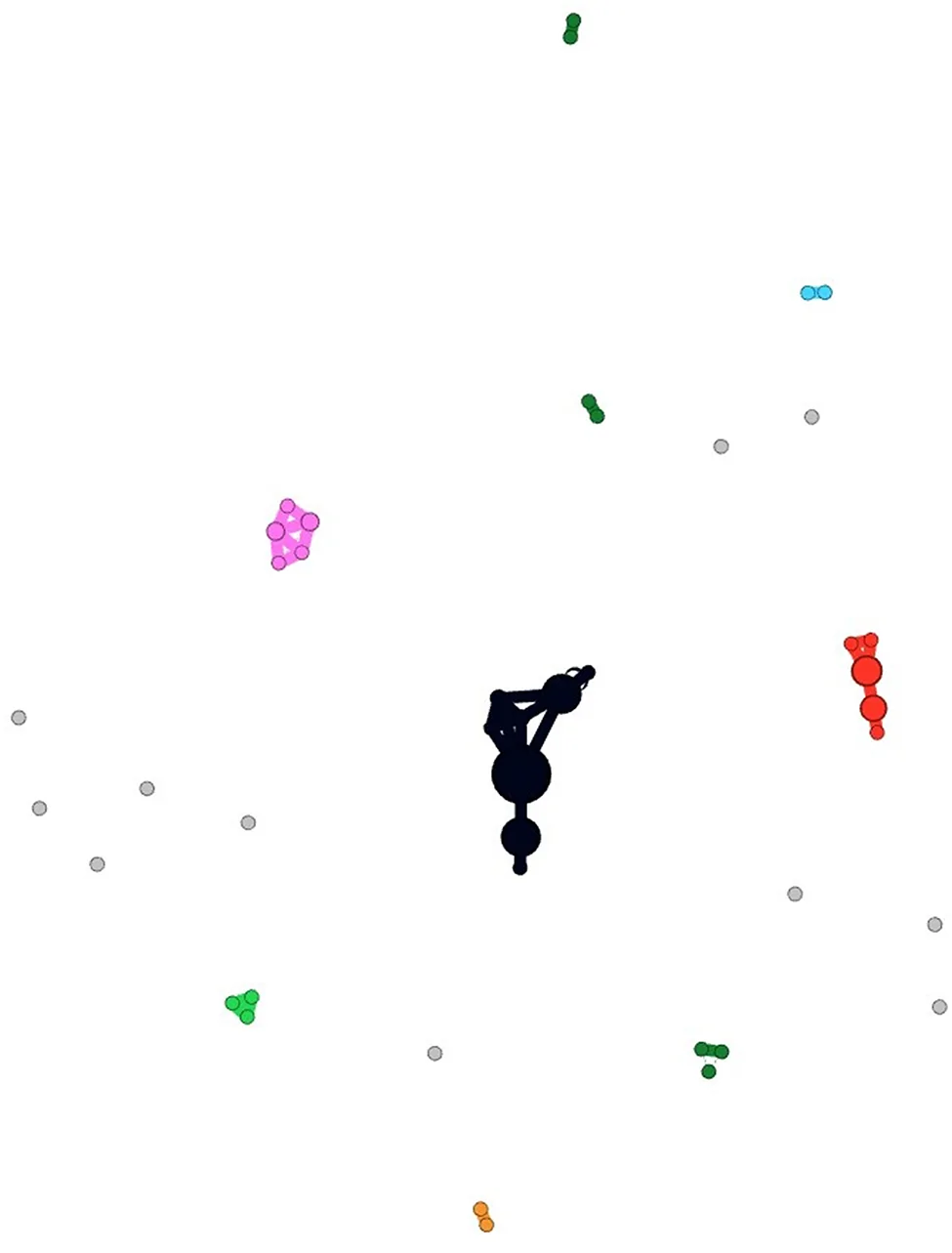
Ego-dependent governance configuration after ego removal. Note: The colours indicate the modules that were identified through the modularity analysis. These were kept constant before and after the ego was removed

Participant 8 is an example of an ego-dependent governance configuration. The network exhibits relatively low *structural integration*, with an edge density of 0.130 and an average degree of 5.591. At the same time, the network shows pronounced modular differentiation, with the highest modularity in the sample (0.579), indicating clearly distinguishable network areas with strong internal connectivity. A high global clustering coefficient (0.717) also suggests strong *local cohesion* within the network (see Fig. 1). *Relational dependency* is particularly pronounced. The ego’s local clustering coefficient is very low (0.030), indicating that its direct contacts are only weakly connected to one another. Combined with the highest ego betweenness centrality in the sample (0.762), this suggests that the focal health professional occupies a pivotal intermediary position between otherwise loosely connected modules. The ego-removal comparison supports this interpretation: although the modules remain intact internally, their connections disappear (see Fig. 2). Therefore, cross-module integration largely depends on the focal actor. This structural pattern is reflected in the participant’s description of coordination within the local governance environment.

*The neighborhood managers and we […] take on*,* in a sense*,* a central role or centralizing function. Not entirely*,* (.) but certainly to some extent. There are also working groups for specific target groups. I think this makes more sense than having a single central coordination structure*.

Overall, this configuration combines cohesive local structures with pronounced relational dependency for integration across modules. Although the participant describes coordination as involving neighborhood managers and topic-specific working groups, the network analysis reveals that connections between modules remain strongly dependent on the focal health professional.

**Fig. 3:**
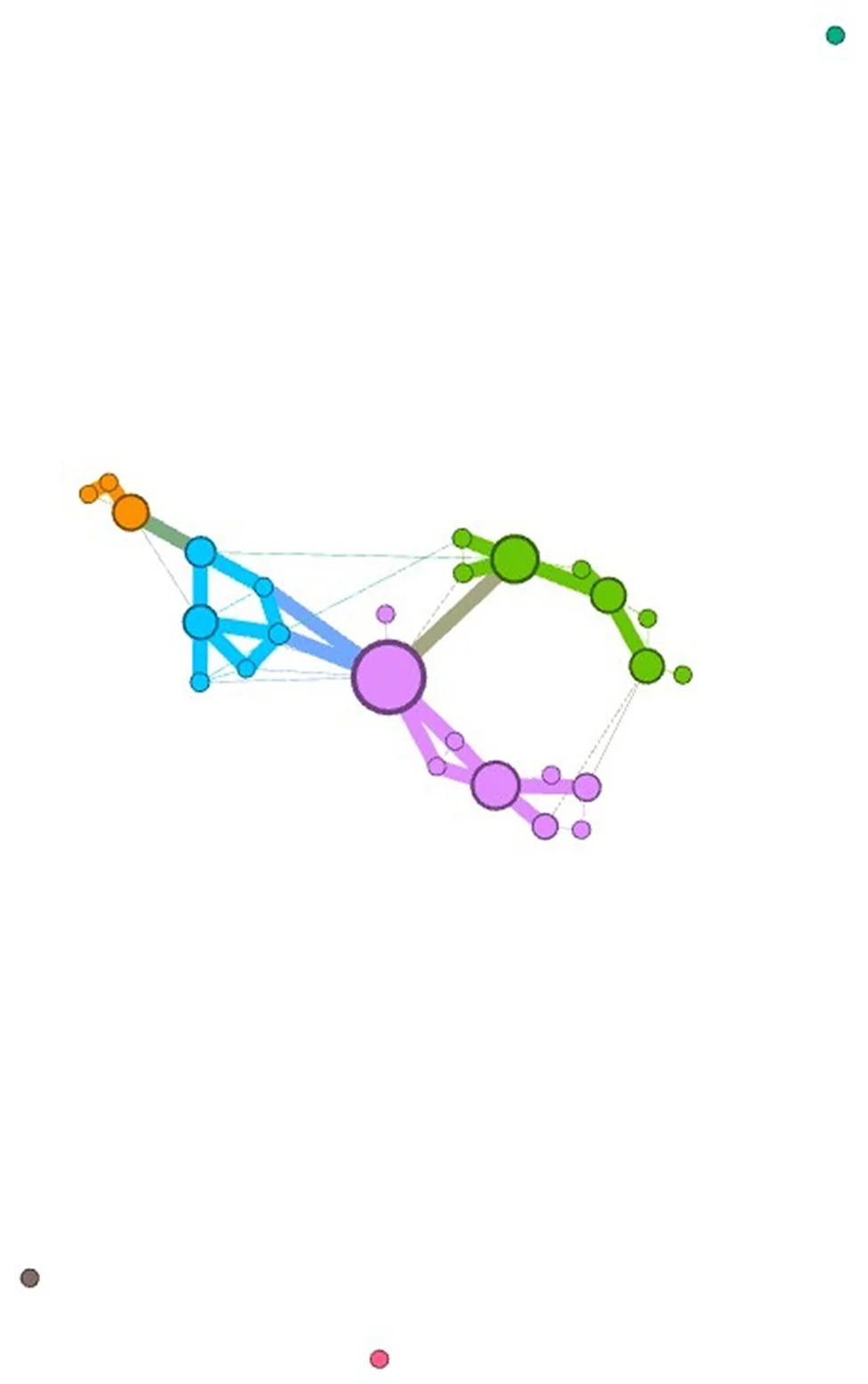
Distributed governance configuration

**Fig. 4:**
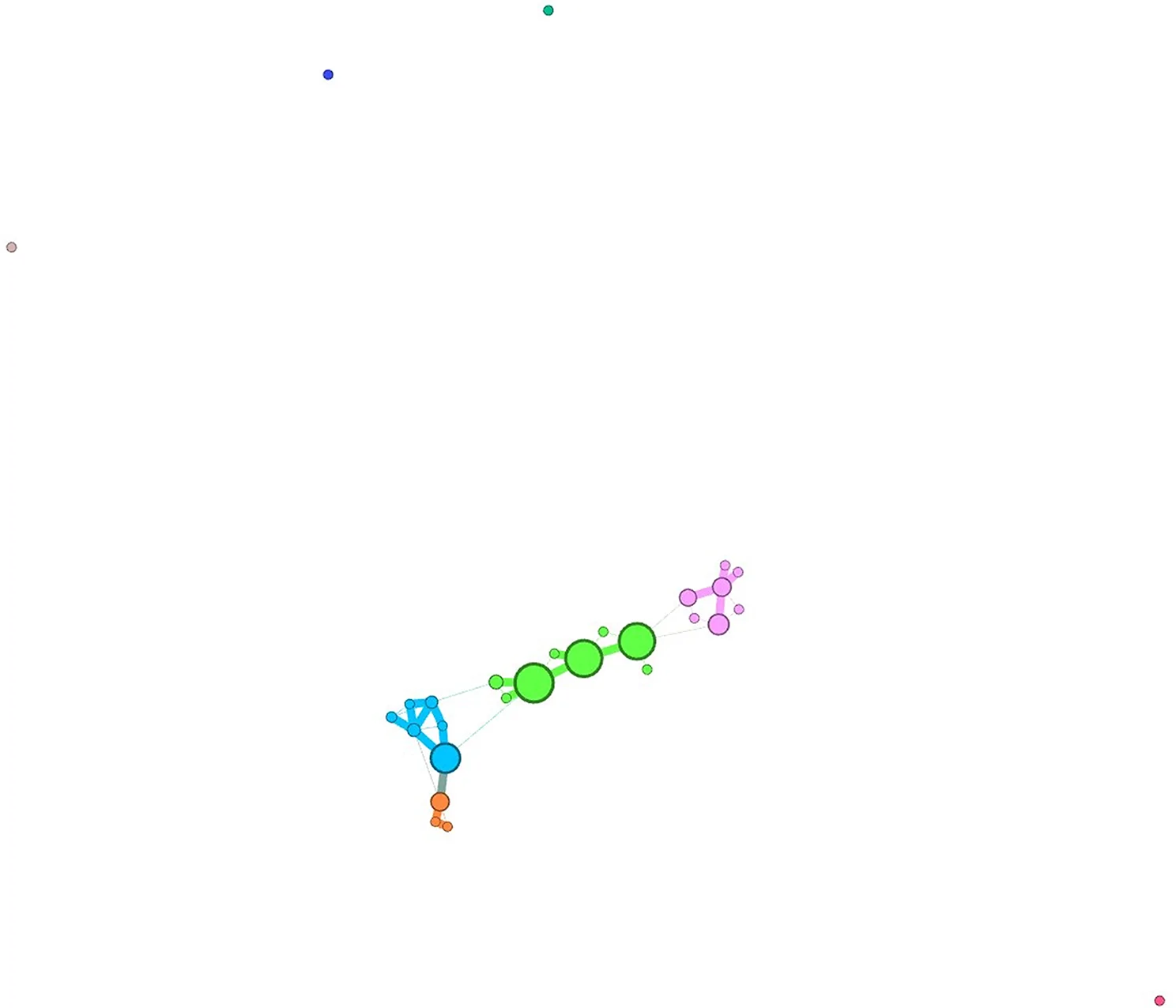
Distributed governance configuration after ego removal

Participant 11 represents a distributed governance configuration. Despite the network’s small size (*n* = 29), *structural integration* is comparatively high, as indicated by an edge density of 0.246 and an average degree of 6.897. *Modular differentiation* is moderate, with a modularity value of 0.482. A global clustering coefficient of 0.556 indicates substantial local cohesion. *Relational dependency* is considerably lower than in the ego-dependent configuration. With an ego betweenness centrality of 0.365, the focal health professional occupies an intermediary position, though they do not dominate connections between network areas (see Fig. 3). This interpretation is reinforced by the ego-removal comparison: after the focal actor is removed, all modules remain connected through alternative relationships. The number of isolated actors increases by only one, from three to four, indicating that the removal of the focal health professional disconnects only one additional actor from the network. Cross-module integration therefore remains largely independent of the health professional and is distributed across multiple relationships (see Fig. 4). This structural pattern corresponds with the participants description of entering and relying on an already established network:

*I’ve joined this network too*,* and there have been people here before me and there will be people here after me*,* so I don’t need to reinvent the wheel. And that’s exactly why*,* um*,* I think you can benefit from the network by simply making use of the structures that are already in place*,* rather than having to start from scratch every time; that just makes work processes run more smoothly.*

Overall, this configuration combines a differentiated, modular structure with distributed, relational integration. Existing relationships provide continuity beyond the focal actor, enabling cross-module connectivity that persists independently of the health professional.

**Fig. 5:**
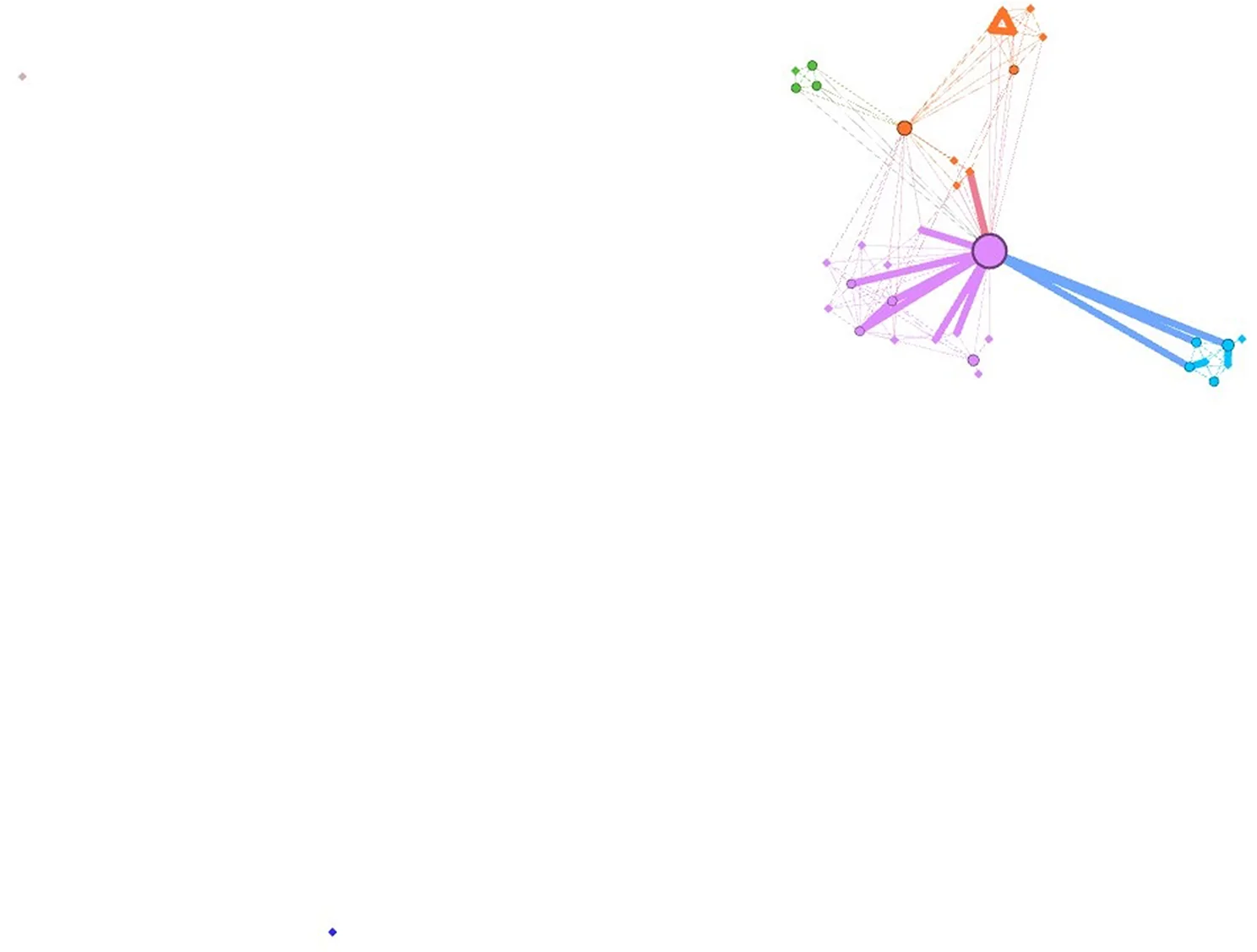
Substitutable governance configuration

**Fig. 6:**
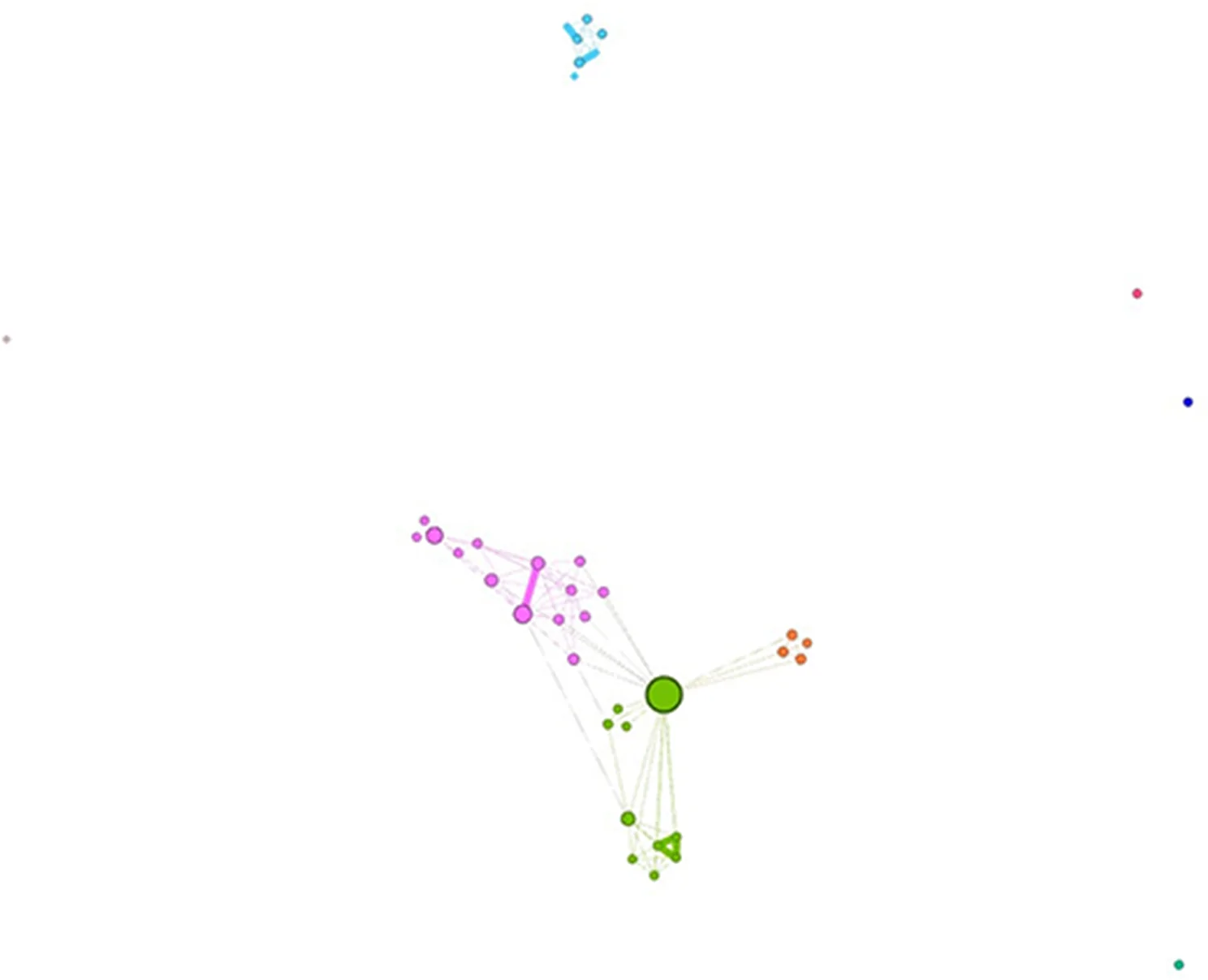
Substitutable governance configuration after ego removal

Participant 5 represents a substitutable coordination configuration. *Structural integration* is particularly high, with the highest edge density (0.303) and average degree (11.800) in the sample, as well as the shortest average path length (2.073). *Modular differentiation* is comparatively low (modularity = 0.419) and *local cohesion* is pronounced, as indicated by a high global clustering coefficient (0.716) and the largest number of triangles (*n* = 66) among the three contrasting cases. *Relational dependency* is present but not concentrated exclusively in the focal health professional. The ego betweenness centrality of 0.495 indicates a relevant intermediary position, while the network also contains alternative coordination centres (see Fig. 5). The ego-removal comparison shows that most modules remain connected, although one module remains internally intact but loses its connection to the rest of the network. Unlike the other substitutable coordination configurations observed in Participants 6 and 7, where another individual actor emerges as the central hub of the remaining network, Participant 5 is distinguished by the central position of the working group ‘Health’ (see Fig. 6). This suggests that substitutability may be supported not only by alternative individual actors but also by collective coordination structures. This pattern is reflected in the participant’s description of both the value and limitations of central coordination:

*It is helpful to have a central point that maintains an overview. That is something that is missing. I also noticed this with neighborhood management. I had imagined that this person would be the one who has the overall responsibility*,* knows what everyone is doing*,* and is in contact with everyone. But that is not the case*,* because the role also includes other responsibilities and may focus more strongly on individual projects rather than being solely responsible for maintaining this broader overview.s*.

Overall, this configuration achieves a balance between high structural integration, local cohesion, and selective relational dependency. While most cross-module connections are redundantly embedded in the network, the integration of individual network areas remains dependent on specific intermediary relationships.

## Discussion

This study investigated which governance structures provide favorable conditions for cross-sectoral coordination in local health promotion networks. The analysis revealed significant structural differences between the examined ego-networks, despite them being located in the same municipality. These differences concerned levels of integration and modular differentiation, local coherence, and the extent to which connections between network areas depended on individual actors. Combining structural network metrics with the ego-removal comparison illustrates that similar coordination tasks can be performed by different governance configurations. The comparative analysis identified three fundamental patterns: person-centered integration, in which links between network areas depend heavily on a single health professional; substitutable coordination, in which alternative individual or collective actors also secure cross-sectoral connections; and distributed integration, in which the connection between network areas is maintained across multiple relationships (see Table 3). The results suggest that sustainably securing coordination capacity is not synonymous with the density of interconnections. For cross-sectoral integration, the structural embedding of mediating functions is equally important.

**Table 3:** Governance configurations and structural patterns across the ego-networks

	Governance configuration	Structural pattern
Ego 1	Ego-dependent integration	Coordination is strongly centred on the ego. Following the removal of the ego, the remaining structure is only loosely connected, consisting of weakly connected clusters, emerging singletons and an isolated cluster. This indicates a substantial dependence on the focal actor for broader network integration.
Ego 2	Substitutable coordination	Although the ego occupies a central hub position, additional star-shaped clusters provide alternative coordination structures. After removal, an organised tree structure with further nodes remains in place.
Ego 3	Substitutable coordination	Coordination is organised around two central hubs: the ego and another healthcare professional. These hubs are connected within a strongly meshed structure. Following the removal of the ego, the second health professional remains as a central hub, ensuring that the network retains substantial internal connectivity.
Ego 4	Substitutable coordination	The ego is the central hub of a partially connected network, to which an additional hub is connected. Once the ego has been removed, a linear structure remains, with only one unconnected module.
Ego 5	Substitutable coordination	Although the ego occupies an important intermediary position within a highly integrated and cohesive network, coordination is not exclusively concentrated in the focal actor. Following the removal of the ego, the Health Working Group continues to act as a collective coordination hub, with most modules remaining connected. However, one module becomes disconnected from the rest of the network.
Ego 6	Substitutable coordination	The ego acts as a central hub, connecting partially linear and moderately to highly meshed substructures. Following the removal of the ego, the school leadership occupies a central position and maintains a significant portion of the network, suggesting the existence of an alternative individual coordination hub.
Ego 7	Substitutable coordination	The network comprises star-shaped clusters connected by chains between areas. Following the removal of egos, school leadership becomes the central hub of the remaining structure, though some actors become isolated.
Ego 8	Ego-dependent integration	Cross-module integration is highly concentrated in the focal health professional. Following the removal of the ego, the modules remain internally cohesive, but lose their connections to one another. This demonstrates their pronounced dependence on the ego for integration across network areas.
Ego 9	Ego-dependent integration	The ego connects several partially and fully meshed clusters within an otherwise egocentric structure. Following the removal of the ego, only one dense sub-cluster remains strongly integrated, while the wider network becomes loosely connected and several actors become isolated.
Ego 10	Ego-dependent integration	The network is strongly centred on the ego. Following the removal of the ego, the network splits into two distinct sections, demonstrating that the focal actor plays a pivotal role in connecting otherwise isolated network areas.
Ego 11	Distributed integration	Rather than being concentrated in the ego, integration is supported by multiple relationships. Following the removal of the ego, all modules remain connected and only one additional actor becomes isolated. This indicates that the overarching network structure largely persists independently of the focal health professional.
Ego 12	Substitutable coordination	The ego occupies a central position, connecting partially meshed clusters. Following the removal of the ego, the network becomes substantially looser, yet still remains connected through a more linear arrangement involving multiple actors. This indicates that the intermediary function of the ego can be substituted through a chain of relationships, as coordination therefore persists without a single dominant alternative hub.
Ego 13	Substitutable coordination	The ego is one of several structurally significant coordination points within a network containing a tree-like cluster and distributed meshing. Following the removal of the ego, the school leadership remains a central hub connected to a meshed sub-network, indicating an alternative coordination capacity.
Ego 14	Ego-dependent integration	The ego acts as a central hub within a mesh-like, tree-structured network. Following the removal of the ego, only a loosely connected residual network remains, with individual actors retaining weak, star-like connections. This indicates substantial structural weakening when the focal actor is absent.

The findings contribute to debates on how local government actors develop the coordination capacity necessary for HiAP. Karlsen et al. [20] show that intersectoral capacity among local authority professionals is related to organizational factors such as job scope, hierarchical position and formal job description, while Wegrich and Stimac [5] emphasize that new coordination tools have limited impact if the underlying coordination culture is neglected. This study complements these perspectives by examining how coordination capacity is structurally embedded within municipal governance networks. It demonstrates that coordination capacity is not solely dependent on a coordinating role with access to resources, but on whether individual mediation is translated into sustainable relational and organizational structures. Local authority coordination capacity thus emerges from the interplay between the scope of action available to coordinating actors and the cultural and institutional conditions of their governance environment. Resources, formal positions and mandates shape the scope for cross-sectoral action, while trust, cooperative orientation and the handling of organizational autonomy shape the conditions under which coordination is developed and maintained. The findings also suggest that these organizational conditions can be reflected in different forms of structural embedding rather than in a single optimal governance arrangement. However, these findings should not diminish the importance of individual coordinators. Cross-sectoral coordination depends on professionals who are willing to bridge organizational boundaries and are committed to health promotion. Brokerage is therefore the starting point, not an alternative, to structural embedding. Sustainable governance capacity emerges when individual brokerage contributes to relationships and coordination structures that become embedded beyond the individual broker.

Previous studies have shown that cross-sectoral cooperation often hinges on the commitment of individuals who initiate collaborations, build relationships, and mobilize support. Meanwhile, organizational and systemic conditions do little to ensure lasting cooperation [4, 21]. The findings show that the strong position held by these individuals can have ambivalent structural consequences. While strong individual brokers can foster integration, they may also create structural dependency if no alternative cross-sectoral links emerge between network areas. Ego-dependent configurations illustrate this ambivalence. While locally coherent domains are well connected internally, their overarching integration largely relies on a single actor. In such cases, the absence of this mediating role affects interconnection rather than internal functionality. Person-dependent brokerage arises from a combination of high centrality and a lack of alternative pathways between network areas. This is particularly relevant in municipal health systems, where coordinating professionals often have to work with limited resources and within time-limited projects, not to mention high staff turnover. Under these conditions, successful individual brokerage can enable cross-sectoral integration in the short term, but not beyond the individual broker. The results also demonstrate that coordination can be embedded in a structure in various ways. In configurations of substitutable coordination, mediating functions are shared between individual actors and a working group acting collectively. This highlights the importance of networks and working groups in local health promotion, even though partial decoupling of network areas indicates that substitutability is gradual rather than complete. Distributed integration represents a distinct form of structural embeddedness. In the case of Ego 11, for example, connections between network areas are primarily maintained through multiple relationships rather than through alternative central hubs. In this form of network governance, mediating functions are less concentrated in individual positions. While both configurations can reduce dependence on specific individuals, they rely on different structural mechanisms. Boundary spanning should therefore not be assessed solely on the basis of individual actors’ ability to bridge organizational or sectoral boundaries [22]. It is also important to consider whether the connections established through boundary spanning are structurally underpinned. Brokerage is thus not merely an individual activity but a governance function whose structural embedding is crucial for the stability of cross-sectoral coordination.

With regard to strengthening local governance capacity, the findings do not support a single centralized or decentralized organizational model for coordination. In line with Provan and Kenis [9], they suggest that institutional coordination should not be confined to one form of network governance. Therefore, institutionalization should encompass more than just the establishment of permanent coordination bodies. Although secure posts, adequate resources, clear mandates and dedicated time for networking are important prerequisites for boundary-spanning, coordinators also need opportunities to foster shared commitment, facilitate joint decision-making and translate coordination into collective action. This requires qualifications in systems thinking, relationship management, and strategic communication, as well as organizational conditions that recognize and support coordination work adequately. These conditions may include collective coordination bodies, alternative coordinating roles, and direct relationships between organizations. Therefore, brokerage should be recognized as a core governance function. The challenge is not to replace individual coordinators, but to create conditions in which the relationships and coordination structures they establish become embedded within the wider governance network and persist beyond the involvement of a single actor.

Future research should adopt a dynamic, multi-level approach to coordination, examining how governance configurations evolve over time in response to changes in municipal health personnel, for example. Longitudinal network studies could clarify whether individual brokerage fosters alternative coordination pathways, and identify which relational investments have lasting structural effects. Further work should also explore the conditions under which coordination becomes ego-dependent, substitutable, or distributed. This could be achieved by combining network analysis with qualitative approaches to understand how organizational positioning, mandates, trust, and coordination cultures shape structural embedding. Conceptualizing brokerage as both a structural position and a professional practice could help identify the competencies and conditions enabling coordinators to establish enduring coordination structures.

### Limitation

Several limitations of this study should be considered when interpreting the findings. Firstly, the sample size was relatively small, primarily due to the time-consuming nature of data collection. Secondly, the study focused on a specific group of health professionals working in municipal settings in Germany. While this approach yielded in-depth, context-sensitive insights, the findings cannot be generalized to other settings. Thirdly, as the study relied on self-reported interview data, participants may have described their networking practices in a socially desirable way. Fourthly, as participation in the study was voluntary, there is potential for selection bias, with more engaged or reflective professionals potentially being overrepresented and influencing the observed network patterns. This could have led to an overemphasis on collaborative behaviors. Furthermore, the cross-sectional design only captures network structures at a single point in time. This means that it could miss the network’s dynamic evolution due to staff turnover or changing collaborations.

Additionally, the methodological limitations of ego-centered network analysis must be acknowledged. This type of analysis relies solely on the interviewee’s perspective, ignoring the views of other network members. However, prior research has shown that the validity of network data generated through naming is relatively high [23]. Furthermore, Marsden [18] demonstrated that egocentric network data can be utilized to approximate specific structural characteristics of entire networks, particularly those based on degree and betweenness centrality.

## Conclusion

This study shows that cross-sectoral coordination in municipal health promotion depends not only on the presence of coordinating actors or the overall connectedness of governance networks, but also on how coordination functions are structurally embedded. Three governance configurations were identified: ego-dependent integration, in which cross-sectoral connections rely strongly on individual coordinators; substitutable coordination, in which alternative individual or collective actors and relational pathways maintain coordination; and distributed integration, in which connectivity is supported through multiple relationships across network areas.

These findings contribute to research on network governance and municipal coordination capacity, demonstrating that brokerage can be embedded in different structural forms with significant implications for dependency on individuals and continuity. Therefore, coordination should be understood as an organizational and structural capacity rather than an ad hoc task performed by individual actors. Although boundary spanners remain important, coordination becomes fragile when integration depends solely on their intermediary position.

To strengthen municipal health promotion and HiAP, adequate resources and a formal mandate are necessary for coordination roles. Similarly, sustained investment in relational infrastructures, collective coordination arenas, and cross-sectoral relationships is needed to reduce dependence on individual brokers. Network work should be recognized as a continuous strategic function requiring time, reflection and institutional support. The central challenge is not to replace individual brokerage, but to create governance conditions in which the relationships and coordination structures that it enables can persist beyond individual actors.

## Supplementary Information

Below is the link to the electronic supplementary material.

Supplementary Material 1

Supplementary Material 2

## Data Availability

The data generated and analysed during this study are subject to ethical and data protection restrictions and are therefore not publicly available. The corresponding author can provide fully anonymised adjacency matrices without any node-level attributes upon reasonable request, for scientific purposes. This is provided that data sharing is compatible with the original consent of the participants, or that additional consent has been obtained where necessary.
