# Supplementary material for: Governance configurations of coordination in municipal health promotion: insights from an egocentric network analysis
Source: BMC Health Serv Res. 2026 Aug 22;26:1176. doi: 10.1186/s12913-026-15347-8 (PMC13508432; doi:10.1186/s12913-026-15347-8)
Supplement: Supplementary file 1 [file 12913_2026_15347_MOESM1_ESM.docx]

Table A1: Module Types and Their Frequency Across Ego-Networks

| Module | Description | Typical Actors | Frequency^[1]^ |
| --- | --- | --- | --- |
| Neighbourhood & Social Space Module | Community-based coordination spaces that link schools, families, healthcare providers and civil society organisations in order to enable local health and education initiatives. | Neighbourhood health professionals  Neighbourhood management  Community centre  Local civil society organisations  leisure and youth centres  family centres | 13/14 |
| Education Module | Formal education settings and early childhood institutions structure everyday learning environments and pedagogical leadership. | School leadership  Teaching staff  School social work services  Early childhood education and care centres  Early childhood educators | 14/14 |
| Professional Coordination and Peer Network Module | Health professionals form peer networks with project coordinators to facilitate the exchange of knowledge, joint planning and strategic alignment across sectors. | health professionals working in schools and neighbourhoods  regional specialists responsible for the mental health of children and adolescents  health department | 12/14 |
| Health Care Service Module | Clinical providers ensure diagnosis, treatment and medical counselling, as well as access to formal healthcare services. | School medical services  Statutory health insurance funds  General practitioners' surgeries  Public pharmacies  School doctor and school nurse | 7/14 |
| Health Promotion & Health Prevention Module | Structured activities that improve physical and mental health through behavioural and environmental prevention, health education and population-based interventions. | Women's / Men´s health organisations  Specialised prevention and health promotion centres  Publicly funded health promotion programmes  Community-based health initiatives run by associations or initiatives  Sports clubs | 7/14 |
| Specialised and Cross Sector Modules | Issue-specific or cross-sectoral clusters do not form part of the core module, but play a complementary role instead. These modules address specific themes, such as nutrition, safety, mobility, and environmental health. | Police  Environmental, mobility, and climate-related initiatives  School nutrition services | Cross Sector: 9/14  Specialized: 3/14 |

1. The module frequency indicates the presence of a module type within each ego-network (n = 14). Each module type was counted once per ego-network, regardless of multiple occurrences. Cross-sectoral modules were double-coded, with one count per category within the same ego-network.
