# Supplementary material for: Governance configurations of coordination in municipal health promotion: insights from an egocentric network analysis
Source: BMC Health Serv Res. 2026 Aug 22;26:1176. doi: 10.1186/s12913-026-15347-8 (PMC13508432; doi:10.1186/s12913-026-15347-8)
Supplement: Supplementary file 2 [file 12913_2026_15347_MOESM2_ESM.docx]

Table A2: Distribution of network modules across ego-networks

|  | Modul |
| --- | --- |
| Ego 1 | Professional Coordination and Peer Network Module |
|  | Education Module |
|  | Cross Sector Health Promotion and Prevention Module / Education Module |
|  | School Nutrition |
| Ego 2 | Cross Sector Neighbourhood / Education Module |
|  | Professional Coordination and Peer Network Module |
|  | Education Module |
|  | Police Modul |
|  | Health Promotion and Prevention Module |
|  | Neighbourhood and Social Space Module |
| Ego 3 | Education Module (Fokus Daycare) |
|  | Professional Coordination and Peer Network Module |
|  | Neighbourhood and Social Space Module (Focus Youth and Integration) |
|  | Education Module (Focus Early Intervention Focus) |
|  | Neighbourhood and Social Space Module (Focus Housing and inclusion) |
| Ego 4 | Neighbourhood and Social Space Module |
|  | Health Care Service Module |
|  | Education Module |
|  | Professional Coordination and Peer Network Module |
| Ego 5 | Education Module |
|  | Neighbourhood and Social Space Module |
|  | Health Care Service Module |
|  | Professional Coordination and Peer Network Module |
| Ego 6 | Health Promotion and Prevention Module |
|  | Neighbourhood and Social Space Module |
|  | Education Module |
|  | Professional Coordination and Peer Network Module |
| Ego 7 | Neighbourhood and Social Space Module (Focus Sport) |
|  | Neighbourhood and Social Space Module (Focus Health Projects / Health Promotion) |
|  | Education Module |
|  | Professional Coordination and Peer Network Module |
| Ego 8 | Neighbourhood and Social Space Module (Focus Sport and seniors) |
|  | Education Module |
|  | Neighbourhood and Social Space Module (Fokus youth) |
|  | Health Promotion and Prevention Module |
|  | Health Care Service Module |
|  | Cross Sector Professional Coordination Module / Health Promotion and Prevention Module |
|  | Professional Coordination and Peer Network Module |
| Ego 9 | Neighbourhood and Social Space Module |
|  | Education Module (Focus family support) |
|  | Education Module (Focus **Pedagogical and Nutrition Support)** |
|  | Professional Coordination and Peer Network Module |
|  | Professional Community Center |
| Ego 10 | Educational Module (Focus teachers) |
|  | Cross Sector Education Module / Health Promotion and Prevention Module |
|  | Neighbourhood and Social Space Module (Focus: Health Care Services and financial Support) |
|  | Education Module (Focus family support) |
|  | Cross Sector Neighbourhood / Professional Coordination and Peer Network Module |
| Ego 11 | Educational Module |
|  | Professional Coordination and Peer Network Module |
|  | Health Care Service Module |
|  | Neighbourhood and Social Space |
| Ego 12 | Neighbourhood and Social Space (Focus Family and Seniors) |
|  | Cross Sector Health Care Service / Health Promotion and Prevention / Neighbourhood Module |
|  | Professional Coordination and Peer Network Module |
|  | Neighbourhood and Social Space (Focus Integration) |
|  | Education Module |
| Ego 13 | Health Care Service Module |
|  | Cross Sector Education Module / Health Care Service Module |
|  | Neighbourhood and Social Space |
|  | Education Module |
| Ego 14 | Neighbourhood and Social Space (Focus Sport and Integration) |
|  | Education Module |
|  | Cross Sector Neighbourhood / Health Care Service Module |
|  | Health Promotion and Prevention Module |
|  | Neighbourhood and Social Space (Focus Community Center) |
|  | Cross Sector Health Care Service / Sport Module |
